# Long-Term Continuous Double Station Observation of Faint Meteor Showers

**DOI:** 10.3390/s16091493

**Published:** 2016-09-14

**Authors:** Stanislav Vítek, Petr Páta, Pavel Koten, Karel Fliegel

**Affiliations:** 1 Faculty of Electrical Engineering, Czech Technical University in Prague, Technická 2, 166 27 Prague, Czech Republic; pata@fel.cvut.cz (P.P.); fliegek@fel.cvut.cz (K.F.); 2 Astronomical Institute of the Academy of Sciences of the Czech Republic, Fričova 298, 251 65 Ondřejov, Czech Republic; koten@asu.cas.cz

**Keywords:** faint meteor shower, meteoroid, CCD camera, image intensifier, image processing

## Abstract

Meteor detection and analysis is an essential topic in the field of astronomy. In this paper, a high-sensitivity and high-time-resolution imaging device for the detection of faint meteoric events is presented. The instrument is based on a fast CCD camera and an image intensifier. Two such instruments form a double-station observation network. The MAIA (Meteor Automatic Imager and Analyzer) system has been in continuous operation since 2013 and has successfully captured hundreds of meteors belonging to different meteor showers, as well as sporadic meteors. A data processing pipeline for the efficient processing and evaluation of the massive amount of video sequences is also introduced in this paper.

## 1. Introduction

Modern image sensors used in astronomy provide high sensitivity and high frame-rates, allowing for the detection of weak and rapidly-changing events in the atmosphere. Among these phenomena are meteors, streaks of light that appear in the sky when an interplanetary dust particle ablates in the Earth’s atmosphere. The study of meteors and meteoroids provides clues about their parent objects: comets [1] and asteroids [2]. The light curve of the meteor contains information about the mass of the original particle. Both the shape of this curve as well as the height interval where the meteor radiates correspond to the structure of the parent meteoroid. It is possible to investigate the above-mentioned and many other properties of meteors using video records. Video data has the advantage that if the meteor is recorded with high time resolution from at least two stations simultaneously, its atmospheric trajectory can be calculated. Moreover, the heliocentric orbit can be determined if we know the exact time of the event, which is common for video observation. It was shown that the properties of systems with image intensifiers enable the detection of meteors with masses down to fractions of a gram.

The multi-station observation of meteors using two or more video systems first appeared in the 1970s [3,4], and became a standard technique for the measurement of meteoroid trajectories. Current networks vary in the number of cameras and observation locations. Video systems within the framework of the SPanish Meteor Network (SPMN) use three cameras at three different locations [5]. The Cameras for Allsky Meteor Surveillance (CAMS) system operates 60 identical narrow-angle field of view (FOV, 30${}^{\circ }$) cameras at three locations [6]. The above-mentioned networks employ low-cost 1/3′′ or 1/2′′ security cameras [7] with a typical spatial resolution of 720 × 564 pixels and a frame rate between 20 and 25 frames per second (fps). Highly-sensitive E2V CCD still cameras [8] or cameras with custom-made electronic shutter systems [9] are also utilized. GigE (Gigabit Ethernet) cameras with 30 fps are used in the framework of the French initiative Fireball Recovery and InterPlanetary Observation Network (FRIPON) [10].

**Figure 1:**
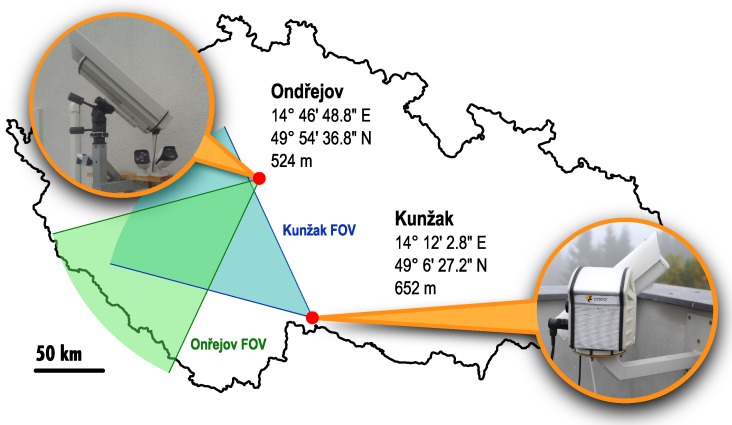
Location of the Meteor Automatic Imager and Analyzer (MAIA) stations. FOV: field of view.

Double-station observation using S-VHS (Super Video Home System) camcorders coupled with image intensifiers started at the Ondřejov observatory about two decades ago [11]. The system had a horizontal resolution of 420 lines per picture, and video data were stored on S-VHS tapes, generally unsuitable for scientific purposes due to the mechanical movement of the tape causing video jitter. The MAIA (Meteor Automatic Imager and Analyzer) system introduced in this paper is a technological successor of the original analog one [12]. It consists of two identical stations placed in Ondřejov and Kunžak, Czech Republic (see Figure 1). The distance between stations is 92.5 km. The Ondřejov camera is aiming at the azimuth of 40${}^{\circ }$, elevation 45${}^{\circ }$, the Kunžak at azimuth of 120${}^{\circ }$, elevation 45${}^{\circ }$.

With spatial resolution close to VGA and temporal resolution up to 61 fps, every single MAIA station produces almost 2 TB of raw video data per night. Since there is limited data storage at each MAIA station, image processing algorithms have to make a decision about the importance of the recorded data during daytime and free up the disk space for the following night. Due to restricted Internet access, the problem of the automatic processing of a massive amount of data has to be solved employing local computing power (e.g., Field-Programmable Gate Array, FPGA, or Graphical Processing Unit, GPU). These techniques make high-performance parallel processing tasks feasible. The computing power of a GPU can reach a thousandfold performance of a standard central processing unit (CPU) with an affordable price. The utilization of a GPU enables a substantial improvement in the performance of astronomical data processing algorithms. At this point, it is worth mentioning related solutions of N-body [13], radio-telescope signal correlation [14], adaptive mesh refinement [15], and gravitational microlensing [16]. These examples mostly focus on complicated numerical and cosmological problems, data mining [17], and the visualization of tera-scale astronomical datasets [18]. Classical image processing problems are addressed to a lesser extent.

The paper is organized as follows. Section 2 gives an overview of the technical properties of the MAIA system. Quality and formal aspects of acquired video data (significantly affecting the design of video processing pipeline) are addressed in Section 3. Section 4 concludes the paper.

## 2. Meteor Automatic Imager and Analyzer

**Figure 2:**
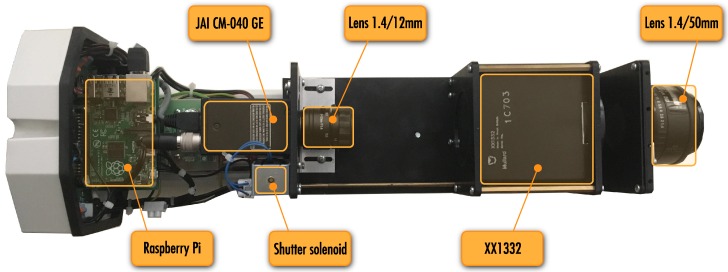
MAIA uncovered.

The design of the MAIA system (see Figure 2) is based on our expertise gained with its previous analog version used in Ondřejov for many years. The electro-optical subsystem of MAIA consists of two main components: a second-generation image intensifier XX1332 and a GigE camera JAI CM-040GE. The image intensifier has a large diameter input (50 mm) and output (40 mm) apertures, high gain (typically 30,000 to 60,000 lm/lm), and a spatial resolution of 30 lp/mm [19]. Since the diameter of the photocathode in image intensifier is 50 mm, and the angle of view for meteor observation should be about 50${}^{\circ }$, then the most suitable focal length of the input lens comes at about 50 mm. The MAIA system uses a Pentax SMC FA 1.4/50 mm—this lens offers the angle of view of 47${}^{\circ }$. Due to the large aperture, a high input signal-to-noise ratio is achieved at the intensifier.

Camera JAI CM-040GE is equipped with a 1/2″ progressive scan CCD sensor. This sensor provides a resolution of 776 × 582 pixels (i.e., 0.45 megapixels), 8.3μm square pixels, and 10- or 8-bit monochrome output. Maximum framerate of 61.5 fps can be increased if needed with vertical binning and partial scan. The exposure time can vary between 54.874 s to 16.353 ms (or preset electronic shutter 1/60 to 1/10,000 in 10 steps) in full frame scan. The camera has high sensitivity of 1.3 lux (on the sensor, maximum gain, shutter off, 50% of peak video level) and a signal-to-noise ratio greater than 50 dB at 0 dB gain setting. The focal length of the camera lens (Pentax H1214-M 1.4/12 mm) was selected to get a perfect match between the output screen of the image intensifier (diameter of 40 mm), the height of the CCD (4.83 mm), and a suitable distance between the camera and the image intensifier (about 10 cm).

The outer housing of the device was selected taking into account weatherproof requirements. The characteristics of the housing are very similar to those required for video surveillance. The body of the housing is made of extruded aluminum, and the end-cover plates of die-cast aluminum. The weatherproof feature is maintained by the rubber gaskets between the cover plates, and three cable glands. The housing is equipped with a heater kit and a sun shield. One-day exposure of sunlight through the fast lens reliably damages the sensitive layer of the image intensifier. Thus, the crucial part of the device is a mechanical shutter fulfilling the function of protection from the Sun. The solenoid opens the shutter for acquisition while a spring ensures that the shutter is closed in the case of power failure. The electro-mechanical design of the solenoid-operated mechanical shutter was a delicate matter. High reliability was required, but electromagnetic compatibility issues also had to be carefully treated, since the image intensifier is prone to electromagnetic interference.

MAIA has a local computer (Raspberry Pi) to handle data transfers between the main computer and the instrument. The data communication between the main computer and the instrument include video stream from the CCD camera, control signals (shutter, heating, local power supply), and environmental data (temperature, humidity). The distance between the device and the main computer is about 10–15 m. A fiber optic cable is used to ensure uncompromising protection of the instrument during thunderstorms.

### 2.1. Electro-Optical Characteristics

**Figure 3:**
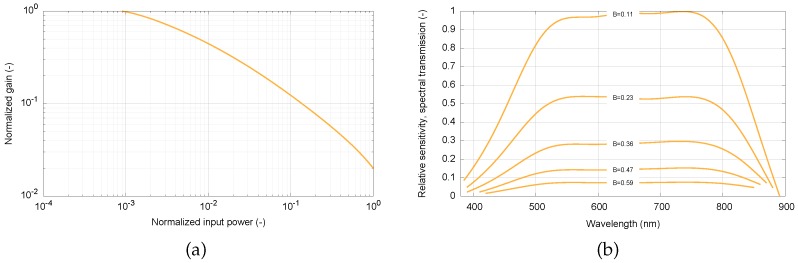
Properties of the MAIA system (**a**) Gain of the intensifier; (**b**) Spectral sensitivity.

The XX1332 image intensifier has a highly nonlinear input–output conversion function as a result of the automatic gain control (AGC). This nonlinearity can be characterized by the dependence of the normalized gain (normalized ratio of the output pixel level in the captured image and the input power of the light, measured at a wavelength of 650 nm) on the normalized input power. The curve describing this dependency is depicted in Figure 3a. The image intensifier’s AGC feature helps to accommodate extremely high dynamic range, and also brings high nonlinearity, which is especially critical for photometric measurements.

The overall relative spectral sensitivity can be seen in Figure 3b. This characteristic takes into account the properties of the input lens, the image intensifier, the camera lens, and the camera itself. There are five curves of the relative spectral sensitivity for several digital values in the output image (B = 1 means white level; i.e., 255 in 8 bpp representation). This result applies to the particular setting of the camera; i.e., electronic shutter set to 1/100 s exposure time, gain of 0 dB, and zero black level. It is evident that the sensitivity is not constant for a chosen wavelength. This is the impact of AGC, as discussed above. The sensitivity is much higher for low-level light conditions in order to achieve sufficiently bright images on the image intensifier’s output screen. However, the spectral dependence of the sensitivity does not change significantly with the variable gain set by the device’s AGC. The FWHM (Full Width Half Maximum) spectral range of the system is approximately 455–845 nm; i.e., slightly shifted to the near-infrared (NIR) domain. This property is crucial, since meteors radiate significantly in this spectral region [20].

### 2.2. Spatiotemporal Characteristics

**Figure 4:**
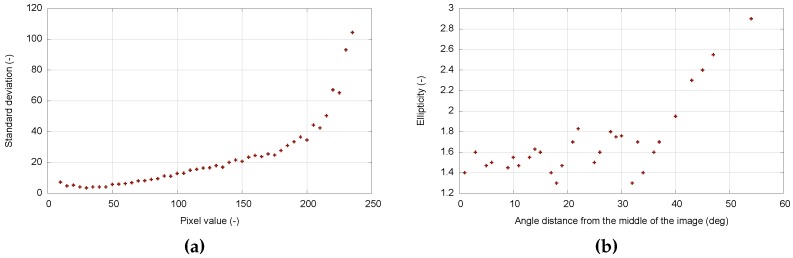
(**a**) Pixel value standard deviations; (**b**) Ellipticity of stellar objects.

Intensified TV systems exhibit a prominent speckle noise component, caused by the intensifier’s AGC. The level of individual bright spots in the video frame fluctuates significantly, while the overall signal level remains roughly constant (i.e., a couple of bright spots increase their level, while the level is decreased for other bright spots). This phenomenon affects conventional image processing algorithms concerning their scalability and performance. Figure 4a shows the dependence of the pixel intensity standard deviation on the pixel intensity value for a video sequence of 100 frames.

**Figure 5:**
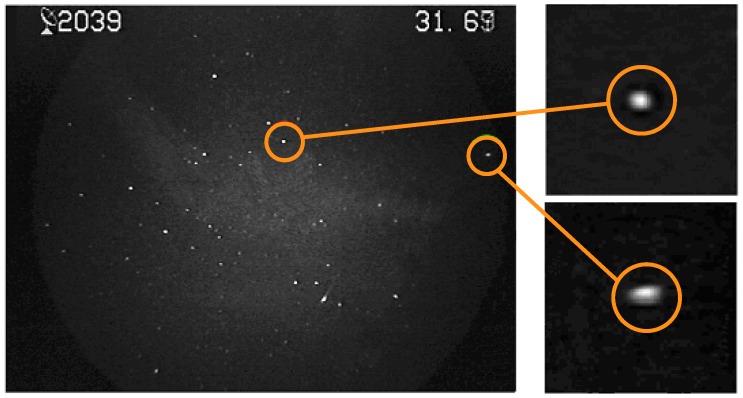
Shift-variant point spread function of the MAIA electro-optical system.

Aside from the high non-linearity discussed in the previous section, MAIA also exhibits features of a shift-variant imaging system. Figure 5 shows the shape of stellar objects in the FOV. It is clearly visible that the object in the middle of the image has a circular shape, whereas objects occurring on the border are heavily distorted. The shape of a stellar object is usually modeled by an asymmetric Gaussian function [21]. Then, the object’s spatial distortion (i.e., its ellipticity) can be described as a ratio of sigma parameters in vertical and horizontal directions. Figure 4b shows the dependence of an object’s ellipticity on the angular distance from the center of the image. The object’s ellipticity plays a significant role in the efficiency of the object detection—for angular distances higher than 40${}^{\circ }$ (i.e., close to the border of the FOV), efficiency decreases significantly.

## 3. Video Data Processing Pipeline

**Figure 6:**
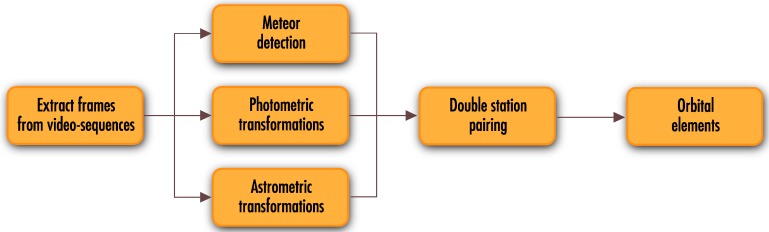
Double-station video processing pipeline.

The primary goal of the MAIA video processing pipeline is to find faint meteor showers in the recorded video sequence. A typical video sequence consists mostly of static stellar objects and noise. There is little or no change between the consecutive frames, due to the high frame rate of the camera. Thus, variations in the image data (e.g., meteors or optical transients) are detectable while using relatively simple algorithms based on comparison via image subtraction. Figure 6 summarizes the essential elements of the proposed video processing pipeline. The double-station system deals with simultaneous observation of the same astronomical events observed from two different locations. Therefore, it is important to ensure synchronous execution of the processes running on both stations. The internal clock of the computer is used as the time authority. An NTP (Network Time Protocol) service is employed to synchronize each computer. Achieved precision of approximately one second is sufficient for the successful identification of meteors recorded in double-station configuration. Precise alignment of the appropriate meteor frames is performed through calculation of the meteor’s atmospheric trajectory.

As discussed in the previous sections, MAIA’s electro-optical characteristics are far from those of an ideal imaging system. Currently, the conventional approach for obtaining the Point Spread Function (PSF) of the space-shift-variant system is based on modeling the wavefront aberrations using Zernike polynomials [22]. An efficient way of bypassing the impact of spatiotemporal fluctuations is described in [23]. The authors employed statistical analysis with nonlinear preprocessing of image intensity using Box-Cox and logarithmic transform. Our pipeline uses a more traditional method based on the detection and classification of the object while taking into account spatial relations between consecutive frames.

### 3.1. Object Detection and Classification

**Figure 7:**
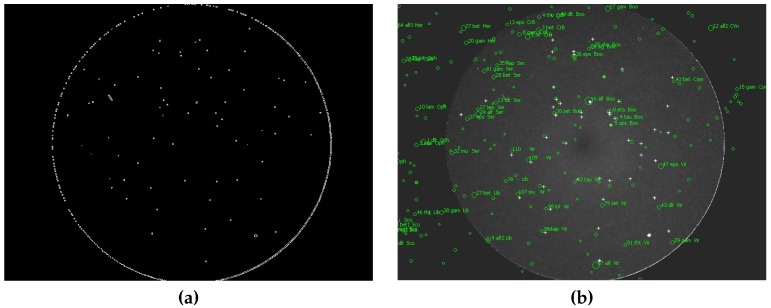
Calibration of the frame. (**a**) Edges detected by Canny filter; (**b**) Identified stars.

A Canny algorithm [24] is used for object detection in the MAIA system. The algorithm detects edges, while static objects are identified as stars (see Figure 7a). The remaining moving objects can be classified as meteors. Linear motion between consecutive frames is then detected for such moving objects. If an object with linear motion is identified within a certain number of consecutive frames (usually at least five frames), then it is classified as a meteor. Finally, the positions of all detected objects are exported into an external text file (MAIA Object File, MOF). Moreover, the video sequence (approx. 80 frames, i.e. 35 frames before and 35 after the event) containing the meteor candidate is saved into a video file and further processed. 

**Figure 8:**
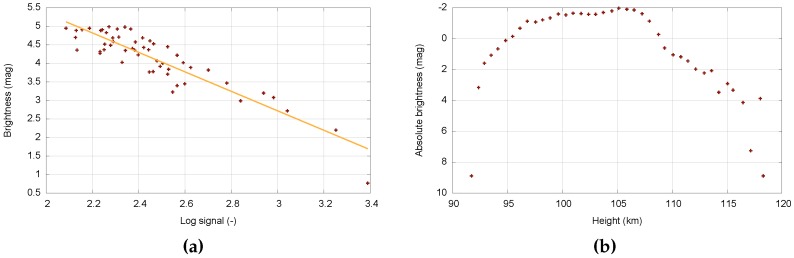
(**a**) Calibration curve; (**b**) Light curve of detected meteor.

The first step in the video processing pipeline is the calculation of the calibration image. The calibration image is obtained as a time average of an odd number of video frames (five frames is usually sufficient). Then, the dark frame is subtracted, and the image is flat-fielded. Stars can be detected using values available in the MOF file. Another approach is based on repeated star detection using the Canny method. The star catalog information is loaded using the previously recorded meteor data (i.e., date, time, aiming point of the camera). Then, the catalog stars can be plotted over the acquired image (Figure 7b). Samples of the detected stars (x, y) and catalog stars (*α*, *δ*) are aligned. Then, parameters of the transformation between both coordinate systems are determined, and the corresponding pairs are identified. The signal of the stellar object is measured as a sum of the pixels in the box surrounding the star. Finally, the calibration curve is constructed (Figure 8a).

**Figure 9:**
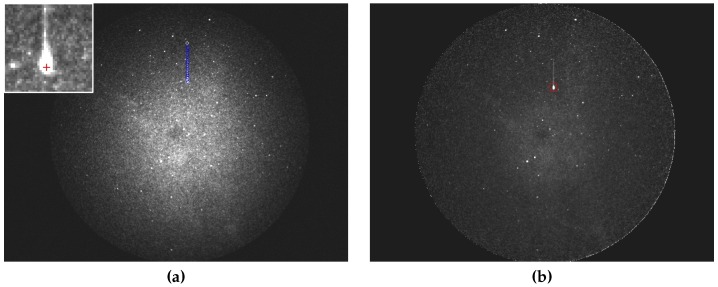
Measurement of meteor parameters. (**a**) Detected Meteor’s path and its magnified detail; (**b**) Meteor signal measured within the box for each frame.

The second step consists of the meteor’s position and brightness measurements. The data stored in the MOF file are used. The particular point can be defined manually, or the MOF positions can be adjusted (Figure 9a). When finished, the boxes around the meteor are set for each frame. The meteor signal is measured as a sum of the pixels within the box (Figure 9b). The brightness of the meteor is determined using the calibration curve. Finally, the light curve of the meteor is calculated (Figure 8b).

### 3.2. GPU Acceleration

The general-purpose CUDA (Compute Unified Device Architecture) GPU is a highly parallel multi-threaded architecture [25]. CUDA became the de facto standard software development kit (SDK) for astronomy computation [26]. One can find numerous studies on the acceleration of image processing for real-time applications, including techniques for real-time moving object detection, a topic related to the subject of this paper. The main bottleneck of GPU acceleration is inefficient data transfer between the host and the device—meaning that implementation of data transfers between the host and the GPU device can negatively affect the overall application performance [27]. The MAIA pipeline solves this issue by batching many small transfers into one larger transfer; i.e., the GPU simultaneously processes more frames.

**Figure 10:**
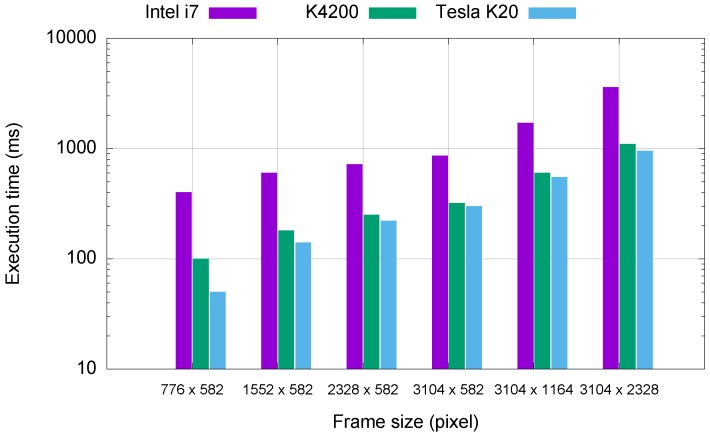
Comparison of the execution time of sequential and parallelized implementation of the Canny detection algorithm.

The most time-consuming operation of the MAIA image processing pipeline is Canny detection. It is used for the detection of objects within particular video frames. This critical part of the pipeline is therefore implemented on the GPU. For the testing, we used video sequences acquired by the MAIA system with a duration of 10 min each (i.e., approximately 36,000 frames at the frame rate of 61 fps). It is worth noting that frame rate is not fully constant and depends highly on the bus workload. The resolution of a single frame is 776 × 582 pixels. Since a pixel takes 2 B of memory (10 b depth, monochrome), one frame requires approximately 1 MB of memory. Consequently, simultaneous processing of more frames brings substantial performance gain. Figure 10 shows the execution times required for processing, depending on the frame size. An image size of 3104 × 2328 pixels means that eight regular input frames are processed simultaneously. Assuming the speed of transfer of about 3000 MB/s between the host and the GPU, this transfer takes around 10 ms, including all necessary data (i.e., convolution kernels, both input and output). We compared execution times of the sequential code written in OpenCV, executed using an Intel Core i7-4790 machine with 16 GB of RAM to parallelized code executed using NVIDIA K4200 and NVIDIA Tesla K40 graphic cards.

The time needed for the processing of a 60 s video sequence on the quad-core CPU is about 100 s. The current version of the GPU-optimized code can process the same sequence in 70 s. Our goal is to process a 60 s video sequence in 60 s or faster (i.e., in real time or faster).

## 4. Conclusions

Automatic double station observations using the MAIA system were carried out continuously during last three years, on each night with good weather conditions. For example, in the year 2015, the camera at Kunžak station was in operation for 202 nights and recorded more than 6500 meteors. The data for the Ondřejov station are slightly lower, since the observational conditions in Onřejov are worse, mostly due to light pollution. Currently, the observations and meteor detection are fully automatic, while the video data processing is semiautomatic, since the operator’s input is still needed.

The comparison of the detection efficiency (which was done for the Perseid campaign) shows that the current MAIA pipeline can detect about 72% of meteors detected by the old system with a S-VHS camera. In the case of the old system, the detection software was usually run several times with parameters tuned manually by the operator. Such an approach is not applicable to the automatic MAIA system. On the other hand, the MAIA system successfully detected some meteors which were not detected by the old system.

The current implementation offers close to real-time video processing; i.e., the processing time is close to the duration of the captured video sequence. Once the development of the MAIA pipeline is finished, it will be released under GPL (General Public License) or a similar license (For information about the current status of the project, please visit http://maia-project.net).
